# New Hosts of The Lassa Virus

**DOI:** 10.1038/srep25280

**Published:** 2016-05-03

**Authors:** Ayodeji Olayemi, Daniel Cadar, N’Faly Magassouba, Adeoba Obadare, Fode Kourouma, Akinlabi Oyeyiola, Samuel Fasogbon, Joseph Igbokwe, Toni Rieger, Sabrina Bockholt, Hanna Jérôme, Jonas Schmidt-Chanasit, Mutien Garigliany, Stephan Lorenzen, Felix Igbahenah, Jean-Nicolas Fichet, Daniel Ortsega, Sunday Omilabu, Stephan Günther, Elisabeth Fichet-Calvet

**Affiliations:** 1 Natural History Museum, Obafemi Awolowo University, HO 220005 Ile-Ife, Nigeria; 2 Department of Virology, Bernhard Nocht Institute for Tropical Medicine, D-20324, Hamburg, Germany; 3 Service des Maladies Infectieuses et Tropicales, Hospital Donka, Conakry, Guinea; 4 Ambrose Alli State University, Ekpoma, Edo State, Nigeria; 5 Department of Veterinary Pathology, Faculty of Veterinary Medicine, University of Liège, Liège, Belgium; 6 Department of Molecular Medicine, Bernhard Nocht Institute for Tropical Medicine, D-20324, Hamburg, Germany; 7 Department of Geography, Benue State University, Makurdi, Nigeria; 8 Fondation John Bost, 24130 La Force, France; 9 Department of Medical Microbiology and Parasitology, College of Medicine, University of Lagos, Nigeria

## Abstract

Lassa virus (LASV) causes a deadly haemorrhagic fever in humans, killing several thousand people in West Africa annually. For 40 years, the Natal multimammate rat, *Mastomys natalensis*, has been assumed to be the sole host of LASV. We found evidence that LASV is also hosted by other rodent species: the African wood mouse *Hylomyscus pamfi* in Nigeria, and the Guinea multimammate mouse *Mastomys erythroleucus* in both Nigeria and Guinea. Virus strains from these animals were isolated in the BSL-4 laboratory and fully sequenced. Phylogenetic analyses of viral genes coding for glycoprotein, nucleoprotein, polymerase and matrix protein show that Lassa strains detected in *M. erythroleucus* belong to lineages III and IV. The strain from *H. pamfi* clusters close to lineage I (for S gene) and between II & III (for L gene). Discovery of new rodent hosts has implications for LASV evolution and its spread into new areas within West Africa.

Lassa fever, a viral haemorrhagic disease, affects 150,000–300,000 people in West Africa, causing up to 5,000 deaths per year^1^. It was discovered in 1969 in Nigeria when American nurses died in Jos Evangel Hospital after a human-to-human transmission^2^. The first case came from Lassa, a village located in Maiduguri region near the border with Cameroon (in present day Bornu state, Nigeria). Shortly after this first outbreak several cases were recorded in eastern Sierra Leone, leading to investigations to identify the virus reservoir among commensal rodents. The Multimammate rat *Mastomys natalensis* was then discovered as a reservoir host of Lassa virus (LASV) in 1974^3^. Till now, there have been a number of reports suggesting other rodents might also be reservoirs of this virus^4^,^5^. However, these reports have not provided convincing evidence through species-level identification of their rodent specimens. More recently, the belief that *M. natalensis* is the sole reservoir of the LASV has been reinforced by studies covering extensive areas of Guinea^6^, Sierra Leone^7^, southern Mali^8^ and in northern Côte d’Ivoire^9^. These recent investigations, identifying their animal specimens via DNA taxonomy, found only *M. natalensis* to be positive for LASV. In the present study, employing molecular techniques both for virus-testing and species-identification of small mammal specimens, we report the first detection of LASV in the rodents *Hylomyscus pamfi* in Nigeria and *Mastomys erythroleucus* in certain localities within Nigeria and Guinea.

## Results and Discussion

### Lassa virus in *Hylomyscus pamfi*, Nigeria

In October 2008 and March 2009, a preliminary screening for arenaviruses was conducted during the process of describing the African wood mouse *H. pamfi* as a new species^10^. Of 10 specimens captured in Kako, southwestern Nigeria, during this period, 4 were LASV positive (Table 1). Partial GP sequences of these strains, labeled Nig09-OSPMH86, 89, 123 and 124, have been used to develop a LASV-specific diagnostic test^11^. At that time, only limited amounts of tissue were available, preventing the isolation of the virus. As these sequences clustered with Lassa lineage I (Lily Pinneo strain) in the partial L phylogeny (Supplementary Figure 2), we decided to go back to the field to catch new animals aiming to obtain a positive specimen that would allow us to perform complete sequencing of the genome. Fortunately, in March 2012, one *H. pamfi* out of 2 captured in Kako was PCR-positive for LASV (Table 1), and the virus was successfully isolated in our BSL4 laboratory. Throughout our sampling in Kako, no *M. natalensis* was PCR-positive.

**Figure 1:**
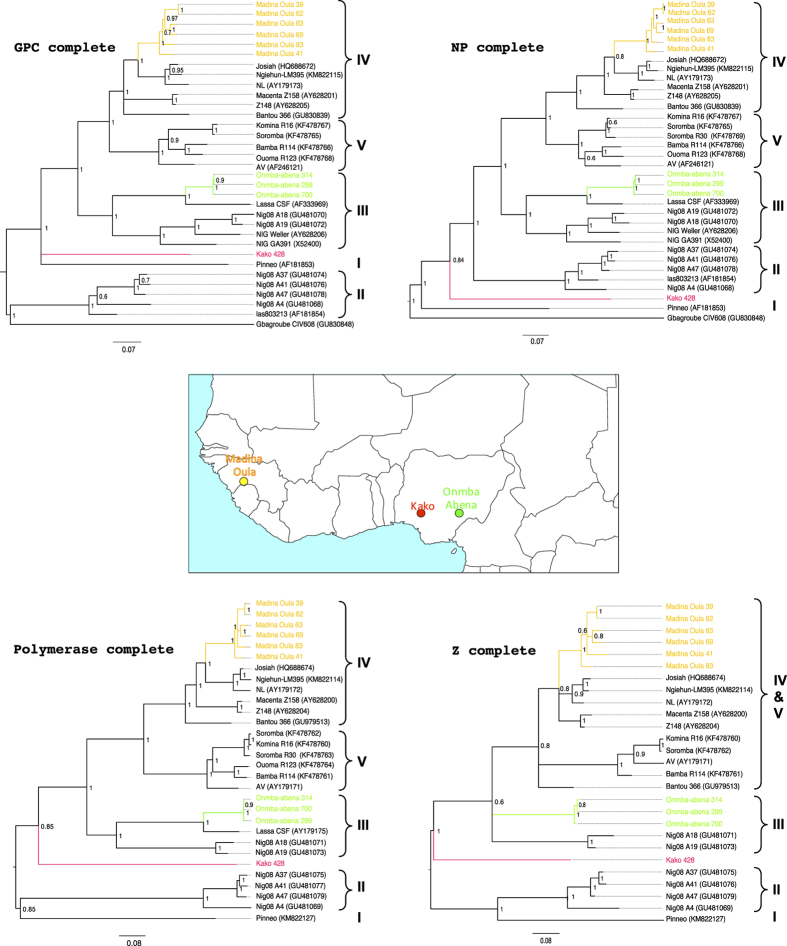
Bayesian phylogenetic analyses based on nucleotide sequences of the complete GP, NP, L and Z segments of LASVs showing the placement of the virus strains isolated in this study in comparison to other sequences representing the members of LASV lineages I–V. Inset is a map of West Africa showing various sites sampled. Different colours (red, green and yellow) of sequences in the phylogenies indicate the geographical origin (in the map) from which the sequences were obtained during this study. Statistical support of grouping from Bayesian posterior probabilities (clade credibilities ≥70%) is indicated at the nodes. Strain names, GenBank accession numbers are indicated on the branches. Scale bar indicates mean number of nucleotide substitutions per site. The map of West Africa was downloaded from http://d-maps.com/carte.php?num_car=752&lang=fr, and then modified using the software EazyDraw v 5.3.0 (http://eazydraw.com).

Phylogenetic analysis of nucleotides of the complete GP and NP genes revealed that the virus strain from Kako clustered close to lineage I (the Lily Pinneo strain), while in phylogenies based on complete L and Z genes the Kako strain clustered between lineages II and III (Fig. 1). Bayesian phylogenies, maximum likelihood and neighbor-joining trees (data not shown) exhibited similar topologies for all genes with high bootstrap support values at most branches. Although we detected no evidence of recombination or reassortment events among various segments of the LASV lineages, phylogenetic analysis based on the amino acid sequences of the L and Z genes clustered the Kako strain differently in a sister relationship but still distant to the singleton Pinneo strain of lineage I or with the members of the lineage II (Supplementary Fig. 1). Collectively, our phylogenetic results (with the Kako strain falling among LASV lineages I-III but not clustering exclusively to any of them) indicate that Kako might constitute a potential lineage of its own. Additional sequences obtained from rodent specimens and possibly also from humans will help to establish this new lineage.

The amino acid differences between the Kako strain and the singleton Lily Pinneo strain is 13.3% for the NP segment, higher than the 12% cut-off suggested by Bowen *et al.*^12^ as a delimitation criterion between LASV and other arenaviruses^12^. However, as increasingly diverse Lassa strains are recovered from humans and rodents (e.g.^7^,^8^,^13^,^14^) the 12% cut-off for designating strains belonging to LASV will most likely have to be revised. In fact, a recent study that generated complete LASV genomes from Nigeria and Sierra Leone^14^ reported high nucleotide sequence variation of up to 32% and 25% for the L and S segments respectively, higher than the findings in Bowen *et al.*^12^. In addition, detection of the Kako strain supports the view that LASV is more diverse than previously thought and is certainly a species complex, with strains emerging that fall within the lineages I-IV but are hosted by rodents other than *M. natalensis*. From an evolutionary perspective, Kako represents the link between LASV and other closely related arenaviruses such as Mobala virus and Mopeia virus discovered in *M. natalensis* in eastern Africa^15^,^16^, and also the more recently detected Gbagroube virus and Jirandogo virus both found in *Mus* spp. in western Africa^17^,^18^.

### Lassa virus in *Mastomys erythroleucus*, Nigeria

Onmba Abena lies within the endemic zone for Lassa fever in Benue state, eastern Nigeria^19^. This village was sampled as part of a larger survey of small mammals for LASV. In 2011–2012, 63 *M. erythroleucus* were trapped in Onmba Abena, out of which 3 were positive for LASV (prevalence 4.76%) (Table 1). All 3 virus strains were isolated in the BSL-4 lab and sequenced for the whole genome. Phylogenetic analyses of the nucleotide sequences from complete GP, NP and polymerase genes show a clustering with Lassa CSF within lineage III, with highly supported nodes (posterior probability = 1, Fig. 1). The same result was obtained at the amino acid level (Supplementary Figure 2), with divergence between Onmba Abena and Lassa CSF of 5.8–6.2% for the NP segment.

In Nigeria LASV lineage III, as detected in humans, circulates in the north central area of the country^20^. The Lassa CSF strain, which falls within lineage III, was also detected in a patient in this area, on the Jos Plateau^21^. As we found CSF-like LASV in *M. erythroleucus* in Onmba Abena, which lies to the immediate south of the Benue River and close to the distribution range of LASV lineage III detected in humans, this suggests that *M. erythroleucus* could possibly also be a host of LASV lineage III in north central Nigeria.

Again, none of the *M. natalensis* in Onmba Abena was LASV PCR-positive. This can be due to the small sample size in October 2011 and October 2012: 4 *M. natalensis* vs. 49 *M. erythroleucus*. After March 2011, the abundance of *M. natalensis* decreased dramatically (a rodent control campaign is suspected) leading *M. erythroleucus* to enter the houses and come into contact with a contaminated environment.

### Lassa virus in *Mastomys erythroleucus*, Guinea

In 1991–92, a spatial survey on LASV seroprevalence in humans showed a high rate in Madina Oula, which is located in the coastal area of Guinea (35%, 59/171)^22^. Various small mammal surveys performed through 1983–2009 show that coastal Guinea is outside the geographic range of *M. natalensis*^23^,^24^. The human and murine data have been compiled and mapped in Supplementary Figure 3. This discrepancy between high human seroprevalence and the possible absence of the reservoir invited us to verify on the field this unusual epidemiological scenario. In May 2014 we captured sixteen *M. erythroleucus* in Madina Oula, while not a single *M. natalensis* was caught. Six animals were LASV PCR-positive (prevalence 37.5%), and phylogenetic analysis of the nucleotide sequences from complete GP, NP, polymerase and Z segments of these BSL-4-isolated strains show that they belong to lineage IV (Fig. 1). In all trees, the Madina Oula sequences clustered to those from Sierra Leone (Josiah, NL and LM395) and forest Guinea (Z148 and Z158) rather than to those from Upper Guinea (Bantou). Phylogenetic analysis of the amino acid sequences shows the same clustering for 3 proteins, except for the GP (Supplementary Figure 1).

Arenavirus antibodies have been detected very recently in *M. erythroleucus* in three other localities within coastal Guinea, with two of these villages being geographically close to Madina Oula in the same prefecture: Kindia^23^. Because of cross-reactivity, IFA tests do not provide conclusive proof that the infection is from LASV but our discovery in Madina Oula points in this direction. Even though the distribution of *M. natalensis* does not extend into Madina Oula, the fact that this village is a well-recognized route and stopover for many travellers makes the human-assisted introduction of LASV in the area likely. Displaced human populations escaping from civil war in neighbouring Sierra Leone (1991–2002) were sometimes settled temporarily in Madina Oula before being moved to proper refugee camps elsewhere. Introduction of LASV-positive *M. natalensis* or LASV-positive humans (reverse zoonosis), may have led to spill-over infections in local *M. erythroleucus* followed by adaptation of the virus and eventual host switching.

*M. natalensis* and *M. erythroleucus* are closely related species, with sympatric populations across West Africa (supplementary Figure 3)^25^. Their role as co-reservoirs of LASV indicates host-switching via recurring spill-over infections, as has been increasingly demonstrated for other virus-rodent models by various authors^26^,^27^. It has been shown that repeated spill-over infections through contact with a donor species can lead to adaptation of a virus and its emergence in an alternate, recipient host^27^. This scenario is supported by serological study mentioned earlier in Guinea^23^ in which Lassa antibodies were detected in *M. erythroleucus* in localities where, on one hand, LASV-positive *M. natalensis* were present (presenting the possibility of spill-over infections) and also in other localities within coastal Guinea, on the other hand, where *M. natalensis* was completely absent.

## Conclusion

Our discovery of alternate rodent reservoirs for LASV in *H. pamfi* and *M. erythroleucus* is quite surprising, considering previous large scale investigations in a country like Guinea that appeared to convincingly dispel this notion^6^. Our results also support the hypothesis that *H. pamfi* and *M. erythroleucus* are full-fledged reservoirs and not just incidental hosts, since 5/12 *H. pamfi* over 4 years in Kako, Nigeria; 3/63 *M. erythroleucus* over 2 years in Onmba Abena, Nigeria; and 6/16 *M. erythroleucus* in Madina Oula, Guinea were positive for LASV.

This study provides increased insight into the evolution of LASV and demonstrates that the virus is more complex genetically and ecologically, maintained by multiple reservoirs. Our investigations have implications for the epidemiology and control of Lassa fever. As the ecology of *M natalensis* (which is mostly commensal) is not exactly the same as that of *H. pamfi* (forest dwelling) and *M. erythroleucus* (more of a generalist) the potential is present for Lassa fever to emerge in fresh niches other than currently exist in West Africa.

## Methods

As part of surveys screening small mammals for Lassa virus, rodents were trapped in Kako, south western Nigeria (N07° 41′ E04 37′); Onmba Abena, eastern Nigeria (N7° 38′ E8° 24′); and Madina Oula, coastal Guinea along the border with Sierra Leone (N9° 52′ W12° 26′) (Fig. 1). Ranging from October 2008 to May 2014, the sampling dates for each locality are detailed in Table 1. Each date represents a session of 3 trapping nights per locality using Sherman live-capture traps. In Nigeria permission to trap rodents in Kako was granted by the Osun State Ministry of Environment and in Onmba Abena by the Gwer West Local government Area, Benue state. In Guinea permission to trap rodents was obtained from the national ethic committee (12/CNERS/12). The methods were carried out in accordance with the approved guidelines. Total RNA was extracted from whole blood (Nigeria) or dried blood on filter paper (Guinea) employing a QIAamp viral RNA Mini Kit (Qiagen Inc.). Extracted RNA was tested for Lassa virus by RT-PCR to amplify the GPC and L genes^11^,^28^. Cytochrome *b* gene sequencing of all Lassa virus-positive rodents unambiguously identified them as *Hylomyscus pamfi* and *Mastomys erythroleucus*. Additionally, all *Mastomys* from Nigeria were sequenced for cytochrome b to exactly distinguish specimens of *M. natalensis* from *M. erythroleucus*. Lassa strains from PCR-positive samples were cultured in the BSL-4 laboratory at the Bernhard Nocht Institute for Tropical Medicine, Hamburg, Germany. The whole genome for each of the Nigerian strains was obtained by Next Generation Sequencing (NGS), and those from Guinea via Sanger technology.

### Complete genome sequencing of Lassa virus strains

The full-length genomic sequence of Nigerian LASV isolates was determined using next-generation sequencing technology. The cell-culture supernatants were filtered through a 0.45-μm filter (Millipore, Darmstadt, Germany) to remove larger debris and bacteria and digested with a mixture of nucleases (Turbo DNase, Ambion, Carlsbad, CA, USA; Baseline-ZERO, Epicenter, Madison, WI, USA; Benzonase, Novagen, San Diego, CA, USA; RNAse One, Promega, Fitchburg, WI, USA) to digest unprotected nucleic acids including host DNA/RNA. Enriched viral particles were then extracted, fragmented, reverse-transcribed, ends repaired, dA-tailed, adaptor ligated and purified. Library preparation was performed using NEBNext^®^ Ultra™ DNA Library Prep Kit for Illumina^®^ (New England Biolabs, Inc. USA). Sequencing was performed using the Illumina MiSeq platform. Reads were trimmed using Skewer v0.1.123^29^ removing adapters and random primers. The sequences were refined in an iterative process of alignment of reads using bowtie2 v2.2.3^30^, pileup using samtools v0.1.19-44428cd^31^ and creating a new reference sequence using FastaAlternateReferenceMaker-GATK v3.3-0-g37228af^32^. Sequence analysis, genomic organization and multiple alignments were performed using Geneious v7.1.8 (Biomatters, Auckland, New Zealand).

The Sanger sequencing of the Guinean LASV strains complete genomes has been performed as follows: RT-PCRs were performed in a thermocycler Seqlab Primus 25 by using the OneStep RT-PCR kit (Qiagen) with a battery of different primers (Supplementary Table 1). Volume reaction was 20 μl: 9 μl water, 4 μl one step buffer, 0.8 μl dNTP, 1.2 μl Fwd primer, 1.2 μl Rev primer, 0.8 μl enzyme and 3 μl RNA. The cycling conditions were: 30 min at 50 °C, 15 min at 95 °C, 45 cycles including 30 sec at 95 °C, 30 sec at 52–55 °C and 1 min at 72 °C. The loop for S segment was obtained with a nested PCR by using Superscript III OneStep RT-PCR (Invitrogen) and Platinum Taq (Invitrogen). Specific outer primers were designed to amplify this fragment: LVSmad 1208+, and LVSmad 1985- (Supplementary Table 1). In round 1, volume reaction was 25 μl: 3.5 μl water, 12.5 μl one step buffer, 1.2 μl Fwd and Rev outer primers, 1 μl enzyme and 5 μl RNA. The cycling conditions were: 30 min at 60 °C, 15 min at 95 °C, 45 cycles including 30 sec at 95 °C, 30 sec at 55 °C and 30 sec at 72 °C. In round 2, volume reaction was 25 μl (16.15 μl water, 2.5 μl PCR buffer, 0.75 μl MgCl_2_, 0.5 μl dNTP, 1.5 μl Fwd inner primer LVS 1303+, 1.5 μl Rev inner primer LVS 1710-, 0.1 μl Platinum Taq, and 2 μl of DNA amplicon). The cycling conditions were: 30 sec at 95 °C and 40 cycles of 15 sec at 95 °C, 15 sec at 55 °C and 30 sec at 72 °C. The loop for L segment was obtained with a semi-nested PCR using the same kits and following the same cycling conditions as for the S loop. Outer primers were LVL 6581a+ and OWS 1+, and inner primers were LVL 6679+ and OWS 1+ (Supplementary Table 1).

### Phylogenetic analysis

The phylogenetic analyses were performed using Bayesian Markov chain Monte Carlo tree-sampling methods based on 2 runs consisting of 4 chains of 1,000,000 with a burn-in of 25% using MrBayes v3.1.2 (http://mrbayes.sourceforge.net/) and parallel maximum likelihood with PhyML v3.0 (http://www.atgc-montpellier.fr/phyml/versions.php) and Neighbor-Joining methods with 1,000 pseudo-replicates. The Akaike information criterion was chosen as the model selection framework and the general time-reversible model of sequence evolution with gamma-distributed rate variation among sites and a proportion of invariable sites (GTR+Γ+I) for nucleotide sequences and Johnes-Taylor-Thorton with gamma-distributed rate variation among sites and a proportion of invariable sites (JTT+Γ+I) for amino acid sequences as the best model. All model selection methods employed, jModelTest 2^33^, Topali v2.5^34^ and MEGA6^35^ detected the same model of sequence evolution that fit best the data sets. All LASV sequences were confirmed as non-recombinant by using the various methods for recombination detection implemented in RDP3^36^ and Phi test in SplitsTree v4.12.3^37^.

All virus and rodent sequences generated by the study have been submitted to GenBank with accession numbers KT992416-KT992450, and KM052324-KM052326.

## Additional Information

**How to cite this article**: Olayemi, A. *et al.* New Hosts of The Lassa Virus. *Sci. Rep.* **6**, 25280; doi: 10.1038/srep25280 (2016).

## Supplementary Material

Supplementary Information

## Figures and Tables

**Table 1:** *Hylomyscus pamfi* and *Mastomys erythroleucus* specimens captured in each site: Number PCR-positive for LASV/Number captured.

	Kako, Nigeria					Onmba Abena, Nigeria				Madina Oula, Guinea
	Oct2008	Mar2009	Oct2011	Mar2012		Mar2011	Oct2011	Mar2012	Oct2012	May2014
*H. pamfi*	2/6	2/4	0/0	1/2	*M. erythroleucus*	0/7	2/20	0/7	1/29	6/16
*M. natalensis*	0/0	0/0	0/6	0/1	*M. natalensis*	0/28	0/2	0/2	0/2	0/0

Results for *M. natalensis* are included on the bottom row to show that no specimen from this species was positive.
