# Supplementary material for: New Hosts of The Lassa Virus
Source: Sci Rep. 2016 May 3;6:25280. doi: 10.1038/srep25280 (PMC4853722; doi:10.1038/srep25280)
Supplement: Supplementary Information [file srep25280-s1.doc]

**Supplementary information for the article**

**NEW HOSTS OF THE LASSA VIRUS**

**AUTHORS**

Ayodeji Olayemi^1^, Daniel Cadar^2^, N’Faly Magassouba^3^, Adeoba Obadare^1^, Fode Kourouma^3^, Akinlabi Oyeyiola^1^, Samuel Fasogbon^4^, Joseph Igbokwe^1^, Toni Rieger^2^, Sabrina Bockholt^2^, Hanna Jerome^2^, Jonas Schmidt-Chanasit^2^, Mutien Garigliany^5^, Stephan Lorenzen^6^, Felix Igbahenah^7^, Jean-Nicolas Fichet^8^, Daniel Ortsega^7^, Sunday Omilabu^9^, Stephan Günther^2^, Elisabeth Fichet-Calvet^2^*

* Corresponding author

**AFFILIATIONS**

1 Natural History Museum, Obafemi Awolowo University, HO 220005 Ile-Ife, Nigeria

2 Department of Virology, Bernhard Nocht Institute for Tropical Medicine, D-20324, Hamburg, Germany

3 Service des Maladies Infectieuses et Tropicales, Hospital Donka, Conakry, Guinea

4 Samuel Fasogbon, Ambrose Alli State University, Ekpoma, Edo State, Nigeria

5 Department of Veterinary Pathology, Faculty of Veterinary Medicine, University of Liège, Liège, Belgium

6 Department of Molecular Medicine, Bernhard Nocht Institute for Tropical Medicine, D-20324, Hamburg, Germany

7 Department of Geography, Benue State University, Makurdi, Nigeria

8 Fondation John Bost, 24130 La Force, France

9 Department of Medical Microbiology and Parasitology, College of Medicine, University of Lagos, Nigeria

**Supplementary table 1:** Primers used for amplification of the Madina Oula strain for the small and large segments. 1, 2, 3, 4, 5 and 6: primers used for the Lassa diagnostic^1,2^.


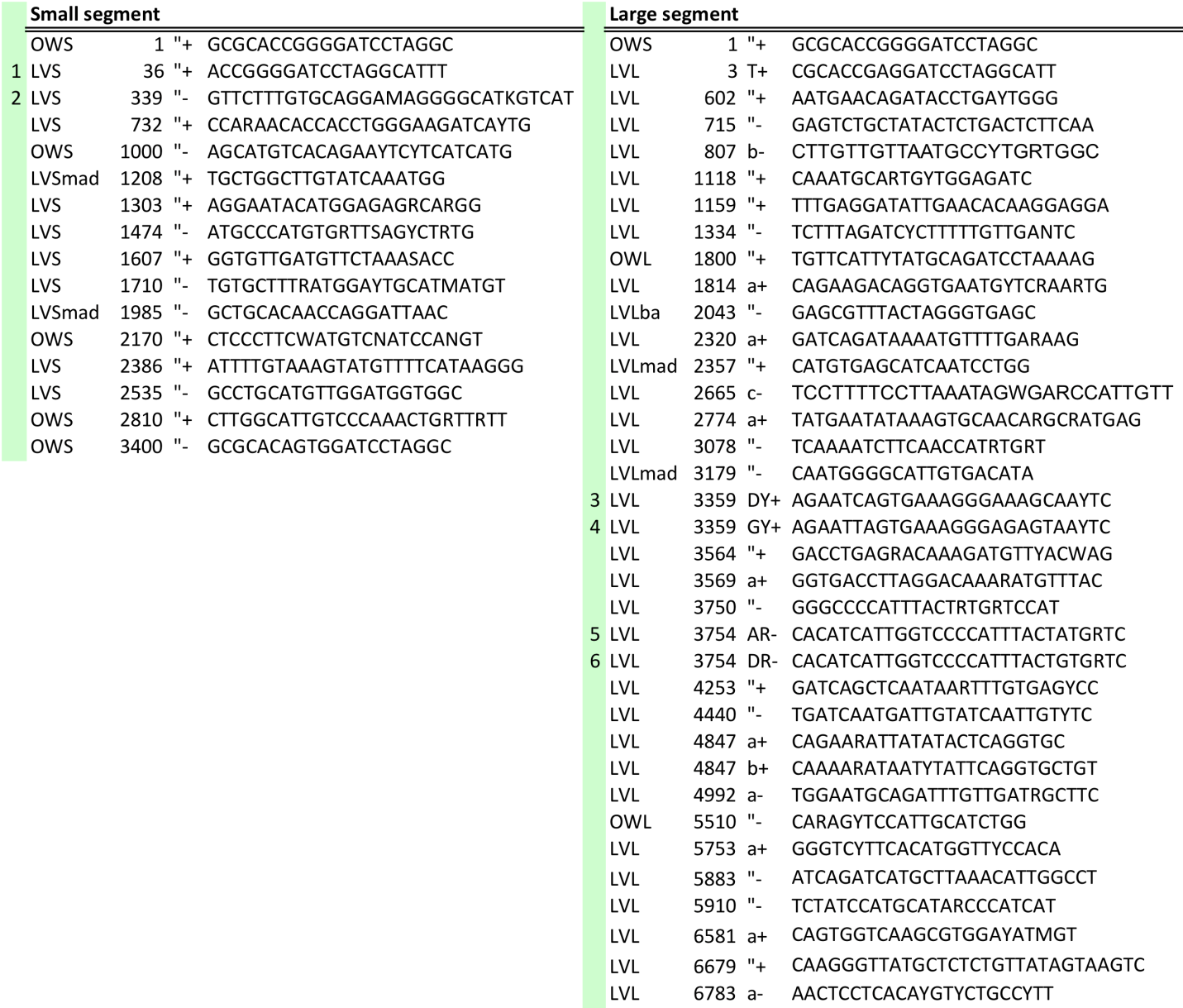


**
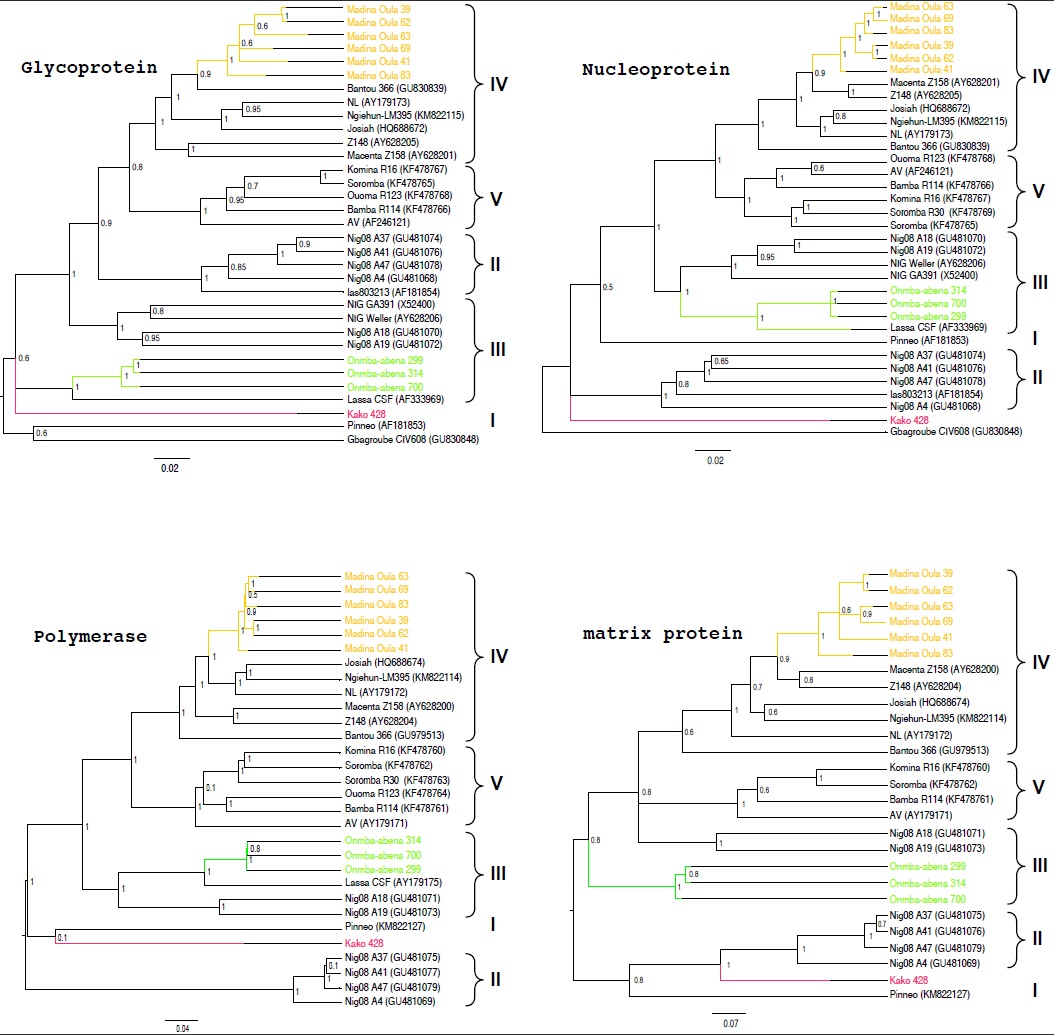
**

**Supplementary Figure 1**: Phylogenetic analyses of amino acid sequences from the GP, NP, L and Z segments showing virus strains isolated from this study in comparison to other sequences representing the Lassa lineages I-V. Scale bar indicates mean number of amino acid substitutions per site.

**
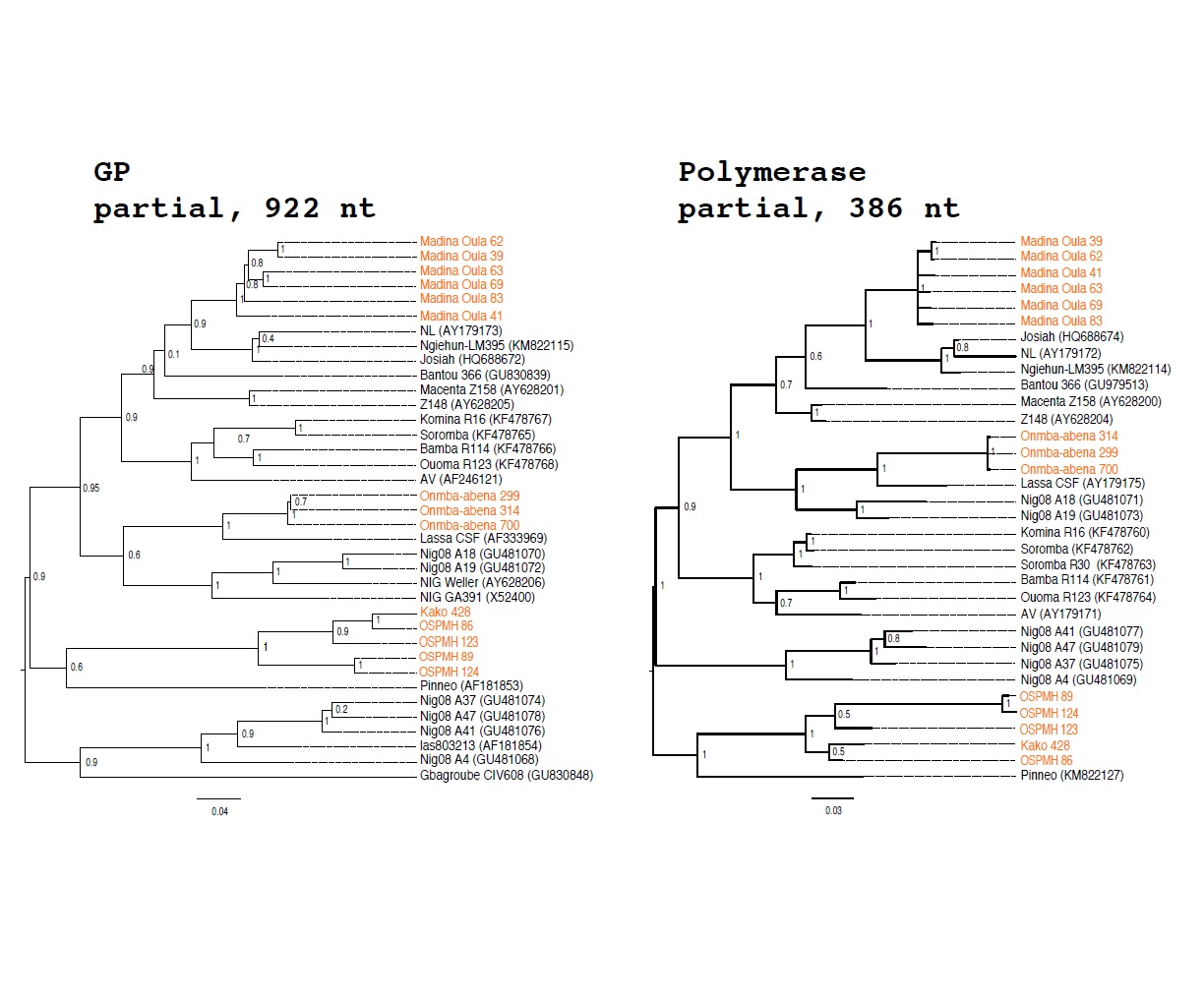
Supplementary Figure 2**: Phylogenetic analyses of the partial GP and L segments including virus strains obtained from Kako in 2008-2009. The trees were inferred by using the Bayesian Markov Chain Monte Carlo method, implemented in MrBayes v3.1.2. Labels in orange are sequences issued from new hosts.


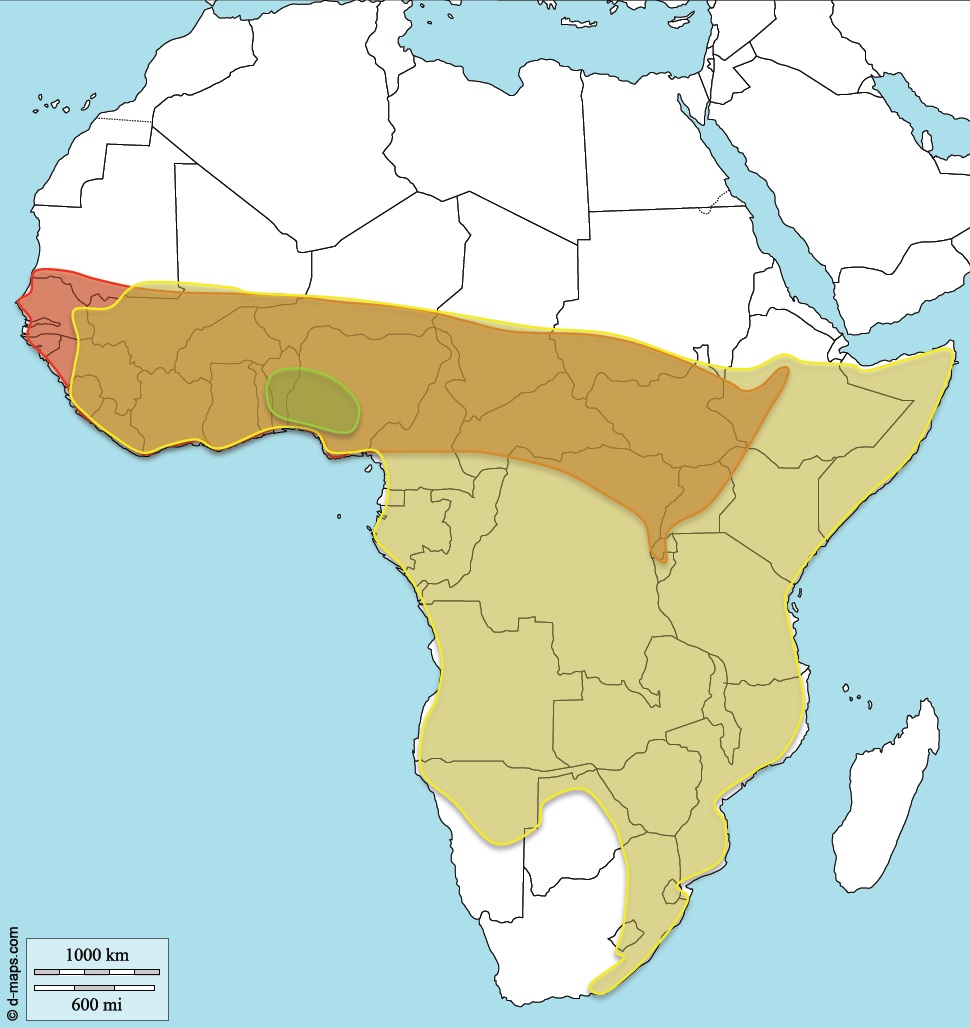


**Supplementary Figure 3**: Distribution of the three rodent reservoirs of LASV in Africa. Red: *Mastomys erythroleucus*^3,4^, yellow: *Mastomys natalensis*^5^, green: *Hylomyscus pamfi*^6^. The map of Africa was downloaded from <http://d-maps.com/carte.php?num_car=736&lang=fr>, and then modified in using the software PowerPoint (v14.1).


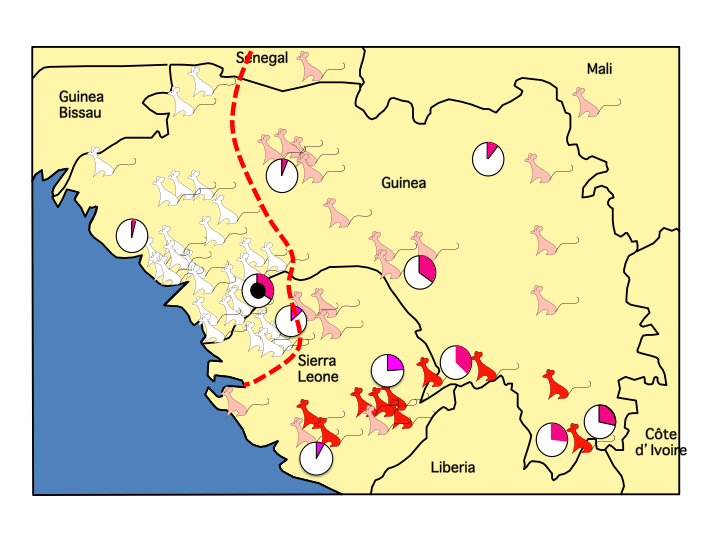


**Supplementary Figure 4**: Distribution of the two species of *Mastomys* in Guinea and Sierra Leone. Mice in red within the map indicate *M. natalensis* only, in white *M. erythroleucus* only, and in pink *M. natalensis* and *M. erythroleucus* in sympatry. The western distribution limit of *M. natalensis* is demarcated by the dotted line. The species identification was done by PCR from 1650 specimens in Guinea and by karyotyping from 387 specimens in Sierra Leone. The pie charts correspond to human Lassa virus seroprevalence with Madina Oula designated by the black dot. The map combines the murine data from Robbins et al. 1983^7^, Duplantier et al. 1997^8^, Lecompte et al. 2006^9^, Lalis et al. 2012^10^, Fichet-Calvet et al. 2009^11^, personal information (Freetown in Sierra Leone), and the human data from McCormick et al. 1987^12^ and Lukashevich et al. 1993^13^. The map was drawn in PowerPoint (v14.1).

References

1 Vieth, S. *et al.* RT-PCR assay for detection of Lassa virus and related Old World arenaviruses targeting the L gene. *T. Roy. Soc. Trop. Med. H.* **101**, 1253-1264 (2007).

2 Olschlager, S. *et al.* Improved detection of Lassa virus by reverse transcription-PCR targeting the 5' region of S RNA. *J Clin Microbiol* **48**, 2009-2013 (2010).

3 Leirs, H. in *Mammals of Africa* Vol. III (ed D.C.D. Happold) 464-465 (Bloomsbury Publishing, 2013).

4 Brouat, C. *et al.* Phylogeography of the Guinea multimammate mouse (*Mastomys erythroleucus*): a case study for Sahelian species in West Africa. *J. Biogeography* **36**, 2237-2250 (2009).

5 Leirs, H. in *Mammals of Africa* Vol. III (ed D.C.D. Happold) 468-470 (Bloomsbury Publishing, 2013).

6 Nicolas, V., Olayemi, A., Wendelen, W. & Colyn, M. Mitochondrial DNA and morphometrical identification of a new species of *Hylomyscus* (Rodentia: Muridae) from West Africa. *Zootaxa* **2579**, 30-44 (2010).

7 Robbins, C. B., Krebs, J. W. & Johnson, K. M. *Mastomys* (Rodentia: Muridae) species distinguished by hemoglobin pattern differences. *Am. J. Trop. Med. Hyg.* **32**, 624-630 (1983).

8 Duplantier, J. M., Granjon, L. & Ba, K. Répartition biogéographique des petits rongeurs au Sénégal. *J. Afr. Zool.* **111**, 17-26 (1997).

9 Lecompte, E. *et al.* *Mastomys natalensis* and Lassa fever, West Africa. *Emerg. Infect. Dis.* **12**, 1971-1974 (2006).

10 Lalis, A. *et al.* The impact of human conflict on the genetics of *Mastomys natalensis* and Lassa virus in West Africa. *PLoS ONE* **7**, e37068, doi:10.1371/journal.pone.0037068 (2012).

11 Fichet-Calvet, E. *et al.* Diversity and dynamics in a community of small mammals in coastal Guinea, West Africa. *Belg. J. Zool.* **139**, 93-102 (2009).

12 McCormick, J. B., Webb, P. A., Krebs, J. W., Johnson, K. M. & Smith, E. S. A prospective study of the epidemiology and ecology of Lassa fever. *J. Infect. Dis.* **155**, 437-444 (1987).

13 Lukashevich, L. S., Clegg, J. C. & Sidibe, K. Lassa virus activity in Guinea: distribution of human antiviral antibody defined using enzyme-linked immunosorbent assay with recombinant antigen. *J. Med. Virol.* **40**, 210-217 (1993).
